# Nanotechniques Inactivate Cancer Stem Cells

**DOI:** 10.1186/s11671-017-2175-9

**Published:** 2017-06-15

**Authors:** Anatoliy N. Goltsev, Natalya N. Babenko, Yulia A. Gaevskaya, Nikolay A. Bondarovich, Tatiana G. Dubrava, Maksim V. Ostankov, Olga V. Chelombitko, Yuriy V. Malyukin, Vladimir K. Klochkov, Nataliya S. Kavok

**Affiliations:** 1 Institute for Problems of Cryobiology and Cryomedicine of the National Academy of Sciences of Ukraine, Pereyaslavska Str. 23, Kharkiv, 61016 Ukraine; 2 Institutes for Scintillation Materials of the National Academy of Sciences of Ukraine, 60 Nauki ave, Kharkiv, 61001 Ukraine

**Keywords:** Cancer stem ells, Ehrlich carcinoma cells, Nanocomplexes

## Abstract

One of the tasks of current oncology is identification of cancer stem cells and search of therapeutic means capable of their specific inhibition. The paper presents the data on phenotype characteristics of Ehrlich carcinoma cells as convenient and easy-to-follow model of tumor growth. The evidence of cancer stem cells as a part of Ehrlich carcinoma and significance of CD44^+^ and CD44^–^ subpopulations in maintaining the growth of this type of tumor were demonstrated. A high (tenfold) tumorigenic activity of the Ehrlich carcinoma CD44^+^ cells if compared to CD44^–^ cells was proven. In this pair of comparison, the CD44^+^ cells had a higher potential of generating in peritoneal cavity of CD44^high^, CD44^+^CD24^–^, CD44^+^CD24^+^ cell subpopulations, highlighting the presence of cancer stem cells in a pool of CD44^+^ cells.

In this study, the ability of synthesized hybrid nanocomplexes, comprising the nanoparticles of rare earth orthovanadates GdYVO_4_:Eu^3+^ and cholesterol to inhibit the tumor growth and to increase the survival of the animals with tumors was established. A special contribution into tumor-inhibiting effect is made by each of its components. Treatment of Ehrlich carcinoma cells with two-component hybrid complex resulted in maximum reduction in the concentration of the most tumorigenic CD44^high^ cells with simultaneous rise in the number of CD117^+^ cells that decreased an intensity of tumor growth by 74.70 ± 4.38% if compared with the control.

## Background

The problem of malignant growth remains one of the most urgent in medicine. In recent decades, there has been some progress in developing new treatments for cancer. This is due to the revision of the classical concept of cancer, and the discovery of cancer stem cells (CSCs), which are capable of unlimited self-renewal and can be identified by a number of phenotypic markers. Most of these cells are resistant to radio- and chemotherapies, causing relapse of the malignant growth and metastasis. There are mastered new methods of anticancer therapy, namely, allowing selectively inactivate tumor cells with minimal damage to normal tissue [1].

The CSCs were for the first time identified and described in 1997 by M. Dick team [2]. The authors investigated an acute myeloid leukemia wherein the subpopulation being 0.01–1% of the total population of cells could cause leukemia when transplanted to the immunodeficient NOD/SCID (nonobese diabetic-severe combined immunodefficiency) mice. These tumor-inducing cells were phenotypically characterized as the CD34^+^CD38^–^. In 2003, M. Al-Hajj and M.S. Wicha succeeded to identify the CSCs in a solid form of human breast cancer (BC) [3]. It has been found that the unseparated population of primary breast cancer exhibited a tumorigenic potential in 100% of cases (10/10) when administered to NOD/SCID mice at a concentration of 5 × 10^4^ cells/mouse. Reducing the concentration of cells administered down to 1 × 10^4^ cells/mouse diminished their tumorigenic activity 4 times (3/12) [3]. CD24^+^CD44^+^ fraction when administered at various doses (2 × 10^4^ down to 100 cells/mouse) did not allow the tumor growth. Herewith, CD44^+^CD24^–^/low subpopulation possessed significantly higher tumorigenic activity, demonstrating the formation of tumors in 100% of cases when administered 10^3^ cells / mouse. The most pronounced ability to form tumors was inherent to the subpopulation of CD44^+^CD24^–^/^lo^ESA^+^ phenotype. Administering to mice just 200 of these cells resulted in the formation of solid tumors in 100% (4/4) 5 months later their injection [3]. These studies were continued by Ponti D. et al., who showed the ability of certain populations of breast cancer biopsy samples to form the mammospheres in vitro in serum-free culture [4]. Most of the cells of the obtained mammospheres were of CD44^+^/CD24^–/low^ phenotype as well as an increased tumorigenic potential in vivo when administered to SCID (severe combined immunodeficiency) mice. The capability of forming tumor in this subpopulation was 1000 times higher versus that for traditionally transplanted line of breast carcinoma MCF7 [4]. However, the authors have shown that only 20% of CD44^+^CD24^–/low^ cells had the capacity for self-renewal. This may be due to heterogeneity of this subpopulation, namely, the presence of additional markers (ESA, ALDH), determining the function of cells and also can be related to the CD44 expression rate. The papers published during the last few years show that the CSCs with a high expression of the marker (CD44^high^) have the highest tumorigenic activity [5, 6]. In orthotopic implantation of 5 × 10^5^ CD44^high^-RAS-transformed and CD44^low^ cells to NOD/SCID mice, it has been found that a subpopulation CD44^low^ possessed low tumorigenicity (tumor was formed in 30% of cases), while the CD44^high^ cells were capable of forming tumors in 100% of cases [6].

Summarizing the published data the differentiation row of subpopulations of breast cancer cells can be represented as follows:

$$ \mathrm{C}\mathrm{D}{44}^{\mathrm{high}}\to \mathrm{C}\mathrm{D}{44}^{+}\mathrm{C}\mathrm{D}{24}^{\hbox{--}}\to \kern0.5em \mathrm{C}\mathrm{D}{44}^{+}\mathrm{C}\mathrm{D}{24}^{+}\to \mathrm{C}\mathrm{D}{44}^{\hbox{--}}\mathrm{C}\mathrm{D}{24}^{+} $$

A number of cells bearing other markers, particularly Sca-1^+^ claims the CSCs stage. The data on a reduction of tumor growth in the Sca-1 knock-out mice indicate in favor of the hypothesis of tumor-initiating role of Sca-1^+^ cells at an early stage of tumorigenesis [7]. Recently, much more attention of the researchers has been attracted not only by CSCs but also the cells making their accessory-regulatory microenvironment. The CD117^+^ cells, which are traditionally detected in a pool of blood stem cells, deserve a particular attention among them [8]. The total population of human breast carcinoma cells comprises the so-called carcinoma-associated fibroblasts of stroma with CD117^+^ phenotype. They support tumor growth, promoting its angiogenesis [9, 10]. An assumption of the presence in Ehrlich carcinoma (EC) population of stem cell, study of tumorigenic potential of CD44^+^ fraction and role of CD117^+^ cells in maintaining the tumor development requires an extra evidence.

Most of the experiments to passage CSCs in vivo were performed in SCID or NOD/SCID mice. These mice do not respond with an immune reaction to xenotransplantation of human cells. The search for adequate and relevant experimental models to study and assess the antitumor activity of various therapeutic agents is in progress. One of them is the in vivo transplanted tumor cell line of EC, which was obtained from a spontaneous breast cancer of mice [11]. However, there are virtually no publications as for subpopulation composition of EC cells and their phenotypic characteristics, presence of CSCs and their importance in maintaining the growth of this type of tumor. Taking into account histogenetic similarity of EC and BC, it can be assumed that in initiation and development of the simulated tumor, the same genes controlling proliferation of cancer cells can be involved as well as similar biochemical pathways leading to the expression of tumor marker proteins. However, the supposition on the presence of CSCs in the EC population and study of their tumorigenic potential needs additional evidence, which was one of the objectives of the present study.

No less urgent problem of current oncology is to find the drugs, not only specifically recognizing but also inactivating the CSCs. The very concept of understanding of the problem was the basis formed in the direction of the moment “theranostics” (therapy + diagnosis) [12]. Within the frames of theranostics, there are developed technological approaches of using the medicines and tools of simultaneous diagnosis and therapy of cancer. One of the directions of theranostics is targeting of gold nanoparticles to tumor site and following photothermal therapy [13]. Other approach to identification of tumor cells is the use of quantum dots, powerful optically contrast agents enabling the monitoring of the tumor in vivo [14]. The ability of the quantum dots to non-invasively visualize the human embryonic stem cells in vivo testifies into favor of their possible biomedical application [15].

Recently bigger attention is paid to nanoluminophors based on dielectric materials and wide zone semiconductors, activated with rare earth elements, namely, nanoparticles (NPs) of rare earth metals (in particular, vanadium and its compounds) [16]. These materials possess a high photostability, large Stokes shift of luminescence, absence of the scintillation effect, and stability of characteristic narrow luminescence bands. Herewith, anti-tumor effects of vanadium compounds are known. So, it has been shown that a vanadium dichloride can significantly inhibit cell proliferation as a result of accumulation in nuclear heterochromatin with subsequent induction of mitotic aberration transient suppression of mitoses, leading to accumulation of cells in late *S* and *G*_2_ phases [17]. Promising for the treatment of malignant tumors can be the use of hybrid nanocomplexes based on rare-earth-based NPs of orthovanadates GdYVO_4_:Eu^3+^ and cholesterol, developed at the Institute for Scintillation Materials of the National Academy of Sciences of Ukraine [18].

The purpose of their creation was to enhance a therapeutic effect of anticancer agents due to the presence in the composition of nanocomplexes having an affinity for the target cell membranes. One is cholesterol which is actively “withdrawn” from the bloodstream by proliferating cancer cells to build the biomembranes. This is facilitated by the presence on the surface of a large number of tumor cells SR-B1 (scavenger receptor, class B type I) and caveolin-1 (Cav-1) receptors, which can bind with the free bloodstream cholesterol [19].

Thus, the aim of this work was to identify the subpopulation composition of EC cells, including those with the signs of CSCs, as well as their tumorigenic activity after pretreatment with hybrid nanocomplexes.

## Methods

The experiments were performed in 8-month-old female Balb/C mice. The mice were kept in standard conditions of the vivarium (room temperature of 20 ± 2 °C, relative humidity 50–70%, light-dark cycle 12:12 h). All experimental protocols were approved by the Animal Ethics Committee of the Institute for Problems of Cryobiology and Cryomedicine of the National Academy of Sciences of Ukraine, Kharkiv, Ukraine, (rec. no. 1 of 23.01.2017) and conformed to the European Convention on the Use of Experimental Animals (Strasbourg, 1986), approved by the First National Congress of Ukraine in Bioethics (Kiev 2004).

### Culturing of EC Cells In Vivo

Ehrlich carcinoma (EC) cells were passaged in the peritoneal cavity (PC) of Balb/C mice. The cryopreserved in ascitic fluid EC cells were used as a primary culture [20]. After thawing, the EC cells were three times re-transplanted in vivo, to the mitigation of an influence of the factors of freeze-thawing and gaining by them of morphological and functional features of native cells [21]. “Stabilized” thereby EC cells were intraperitoneally injected in a dose 3 × 10^6^ cells/mouse in 0.3 ml saline and cultured for 7 days in vivo. After 7 days, the experimental animals were removed from the experiment under light ether anesthesia. Ascitic fluid from PC was taken by a syringe through a needle of 2.69-mm inner diameter and placed into 10 ml measuring tube. Absolute cell number was determined by magnifying the volume of the accumulated in peritoneal cavity ascitic fluid (ml) with the number of EC cells counted in the Goryaev chamber. An increase in total number up to 35.00 × 10^7^ EC cells in PC of mice to day 7 was a criterion of carcinoma development [21]. In future, the very cells served as the object of study.

### Phenotypic assessment of EC subpopulations

It was performed with a flow cytometer “FACS Calibur” (“Becton Dickinson,” USA) using monoclonal antibodies (US “BD Biosciences”) to CD44 (FITC) (no. 553133, clone IM7), CD117 (FITC) (no. 553354, clone 2B8) and Sca-1 (FITC) no. 553333, clone E13-161.7), and CD24 (PE) no. 553262, clone M1/69) according to the manufacturer’s instructions. As a control, the samples with adding nonimmune FITC- and PE-labeled monoclonal antibodies of the same isotypes (“BD Biosciences”), no. 553988, clone A95-1 and no. 553989, clone A95-1), as antibodies to the tested marker were used. An immunophenotypic double staining was performed using CD44 (FITC) and CD24 (PE) monoclonal antibodies. The cells with an average fluorescence of CD44-marker higher 10^3^ (according to a logarithmic scale) were referred to CD44^high^ subpopulations. Recording and analysis of the results were performed with “WinMDi 2.9” software (Joseph Trotter, La Jolla, USA).

### Separation of CD44^+^ Fraction of EC Cells Using Immunomagnetic Sorting

Keeping in mind that the CSCs with a high expression level of the CD44 marker (CD44^high^), comprised by heterogeneous population of CD44^+^ cells, possess the highest tumorigenic activity, they were isolated from a total EC population with a magnetic sorter (BDTM Imagnet). To isolate CD44^+^ faction, there were used primary unlabeled monoclonal antibodies for marker CD44 (BD, 558739) and secondary Mouse IgG1 Magnetic Particles-DM (BD, 557983) according to manufacturer’s protocol. Separation purity for CD44^+^ cells from total EC population was 90%.

### Determination of tumorogenic activity of cells of total population and those of isolated  CD44^+^ and CD44^–^ - EC fractions

**Fig. 1:**
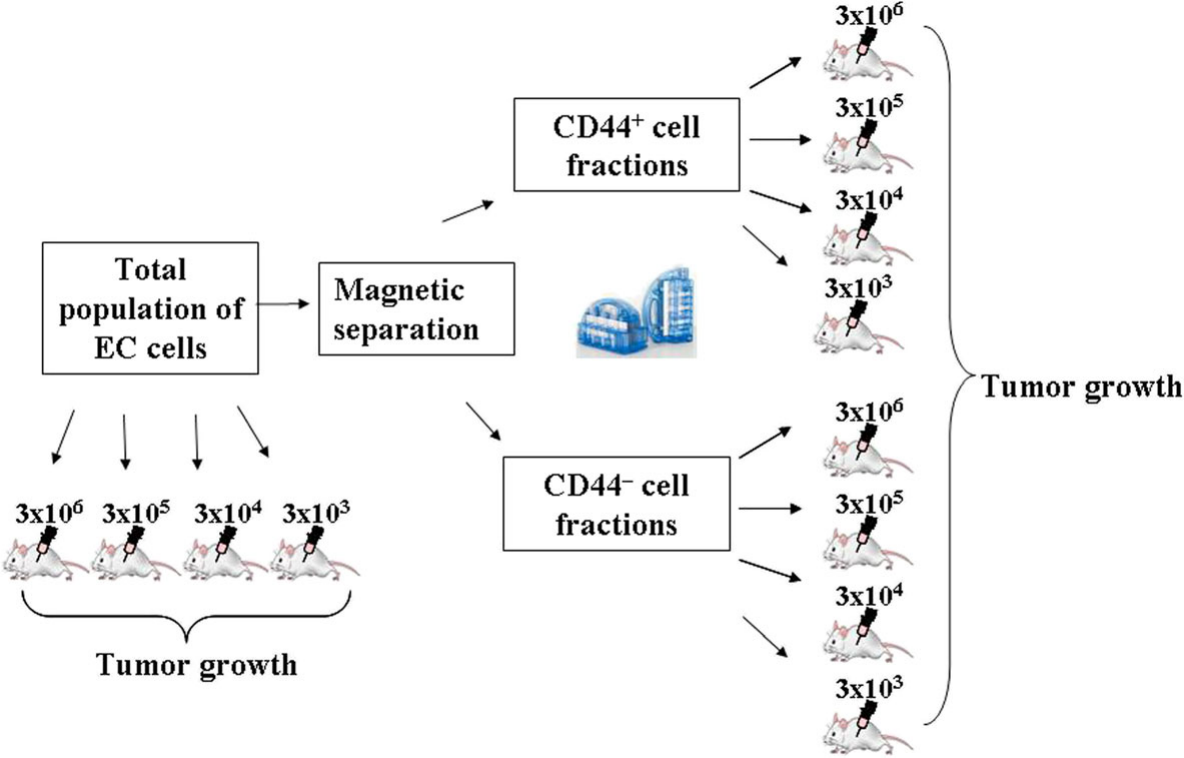
Experimental design when comparatively analyzing the tumorigenic ability of total population and isolated CD44^+^ and CD44^–^ EC fractions

Tumorigenic ability of total population and isolated CD44^+^ and CD44^–^ EC fractions  was comparatively analyzed by the described above method of culturing in vivo. The experimental setup is shown in Fig. 1.

In the first set of experiments, we evaluated the tumorigenic ability of total population and isolated CD44^+^ and CD44^–^ EC fractions when administering them to animals in a standard dose used for EC initiation (3 × 10^6^ cells in 0.3 ml saline).

Animals were divided into the following groups (*n* = 10):

- Group 1.1–administering of total population of EC cells (3 × 10^6^ cells/animal)
- Group 2.1–administering of CD44^+^ fraction of EC cells (3 × 10^6^ cells/animal)
- Group 3.1–administering of CD44^–^ fraction EC cells (3 × 10^6^ cells/animal)

In 7 days after the inoculation in each of the experimental groups, a total number of cells in PC of animals was counted, phenotypic characteristics of cells were assessed (as described above), and CD44^high^/CD117^+^ ratio of EC cells determined as the ratio of CD44^high^ percentage to CD117^+^ cells [22]. Proliferative potential of cells of total EC population and isolated CD44^+^ and CD44^–^ fractions was estimated basing on the data as follows: multiplicity factor (MF) of cell population surplus during culturing time, *M* = N/N_0_; and time doubling (TD), TD = (log_2_2)* *t*/[log_2_ (*N*/*N*_0_)], where *t* is the time of cell culturing (h), *N* is the number of cells at *t* time; *N*_0_ is initial cell number [23].

In the second set of experiments, there was an estimated minimum dose of the administered cells of total population and isolated CD44^+^ and CD44^–^ EC fractions, inducing tumor growth. Total cell suspension and isolated CD44^+^ and CD44^–^ EC fractions were intraperitoneally administered to mice at the doses of 3 × 10^6^, 3 × 10^5^, 3 × 10^4^, and 3 × 10^3^ cells per mouse in 0.3 ml saline and cultured for 7 days in the PC.

Animals used in this set of experiments have been divided into the following groups (*n* = 10):

- Group 1.1–administering of total population of EC cells (3 × 10^6^ cellsanimal)
- Group 1.2–administering of total population of EC cells (3 × 10^5^ cells/animal)
- Group 1.3–administering of total population of EC cells (3 × 10^4^ cells/animal)
- Group 1.4–administering of total population of EC cells (3 × 10^3^ cells/animal)
- Group 2.1–administering of CD44^+^ fraction of EC cells (3 × 10^6^ cells/animal)
- Group 2.2–administering of CD44^+^ fraction of EC cells (3 × 10^5^ cells/animal)
- Group 2.3–administering of CD44^+^ fraction of EC cells (3 × 10^4^ cells/animal)
- Group 2.4–administering of CD44^+^ fraction of EC cells (3 × 10^3^ cells/animal)
- Group 3.1–administering of CD44^–^ fraction EC cells (3 × 10^6^ cells/animal)
- Group 3.2–administering of CD44^–^ fraction EC cells (3 × 10^5^ cells/animal)
- Group 3.3–administering of CD44^–^ fraction EC cells (3 × 10^4^ cells/animal)
- Group 3.4–administering of CD44^–^ fraction EC cells (3 × 10^3^ cells/animal)

In every experimental group, a total number of cells in PC and that of animals with ascites development were determined 7 days later after the EC inoculation.

### Synthesis of Nanocomplexes

Hybrid nanocomplexes containing spherical nanoparticles (NPs) (of 2–3 nm diameter) at a concentration of 1.30 g/l and sheep cholesterol in concentration of 0.55 g/l (“Acros organics”, Belgium) were synthesized at the Institute for Scintillation Materials of the National Academy of Sciences of Ukraine (Kharkiv) as reported [18]. NPs based on orthovanadates of rare earth elements GdYVO_4_:Eu^3+^ of spherical shape in 1.30 g/l concentration were prepared as described [24]. Aqueous colloidal solutions based on orthovanadates have been purified of impurities by dialysis using the membranes “Cellu Sep H1” 3.5 KDa.

**Fig. 2:**
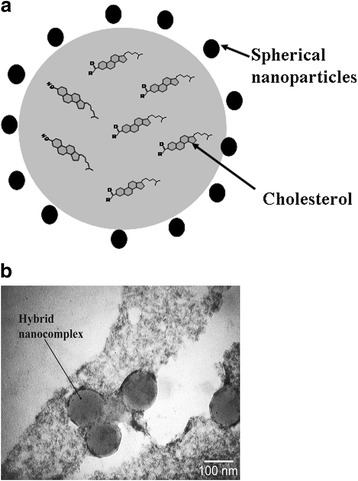
Hybrid nanocomplex: **a** schematic representation and **b** transmission electron microscopy photomicrography of hybrid nanocomplexes, procured from cholesterol aqueous solution placed on carbon network

In hybrid nanocomplex, the negatively charged NPs are localized along the periphery of cholesterol particles due to van der Waals and hydrophobic interactions. The NPs stabilize the nanocomplexes via electrostatic interactions. The sizes of the synthesized nanocomplexes do not exceed 100 nm. In addition, the NPs exhibit antioxidant properties and are not subjected to oxidation. This fact contributes to the rise in resistance of aqueous dispersion of cholesterol in relation to the reactive oxygen species. Schematic structure of hybrid nanocomplex is shown in Fig. 2.

To record an accumulation of hybrid nanocomplexes in cells during the in vitro studies, hydrophobic fluorescent dye 1,1′-dioctadecyl-3,3,3′,3′-tetramethylindocarbocyanine perchlorate (DiI) could be additionally introduced into of cholesterol aqueous dispersion, allowing in local luminescent spectroscopy to evaluate the dynamics of integration of the complex into a cell membrane by the ratio of the monomer––“J-aggregate” luminescence bands [25]. Our earlier studies have shown that hybrid nanocomplex is able to be integrated not more than into 10% of cells of total EC population and virtually to all the cells of isolated CD44^+^ fraction having the highest carcinogenic potential. This allows the use of hybrid nanocomplex in this modification (NPs + cholesterol + DiI) as a method of identifying local accumulation of nanocomplexes in cancer cells [26, 27].

### Pre-Treatment of EC Cells with Nanomaterials

Total suspension of EC cells with hybrid nanocomplexes or NPs was incubated in a solution of 5% glucose (“Infusion” CJSC, Kyiv) at room temperature for 3 h. Such an incubation time was previously found as an optimal for binding nanocomplexes to cells [26].

The following variants of EC cells pre-treatment with nanomaterials were tested:

- Option 1–900 μl of EC cells (1 × 10^7^) 100 μl of spherical NPs (1.3 g/l) were added.
- Option 2–900 μl of EC cells (1 × 10^7^) 100 μl of hybrid complex (spherical NPs (1.3 g/l) + cholesterol (0.55 g/l)) were added.

The control was the cells of EC total population, which were incubated in 5% glucose solution with no treatment with nanocomposites. The number of animals in each experimental group was not less than 20.

After incubation, the EC cells of all the tested groups were washed three times with saline (1:1) by centrifugation (10 min at 300 g).

Intensity of EC development after pre-treatment with nanomaterials was evaluated by intraperitoneal injection in a dose 3 × 10^6^ cells in 0.3 ml saline. In 7 days after EC cell inoculation in all the studied groups, there were determined:

- A total number (TN) of EC cells in peritoneal cavity.
- Inhibition rate (Ri) of EC growth according to the formula Ri = (TN (c) – TN(e)): TN (c) × 100%, where TN (c)––total number of EAC cells in PC of the control group, TN (e)—total number of EAC cells in PC of the experimental group.
- Growth rate (Rg) of EC was calculated using the formula Rg (e) = Rg (c) – Ri, where Rg (e)—growth rate of tumors of experimental group of animals; Rg (c)—growth rate of tumor of the control group, Ri—inhibition rate of EC growth in experimental group of animals; inhibition rate of EC growth in control was taken as 100%, at that, there was no inhibition of EC growth.
- CD44^high^/CD117^+^ ratio (ratio of CD44^high^ percentage to CD117^+^ cells).
- Animal survival was assessed to day 20 after intraperitoneal injection of untreated and treated with all types of nanocomposites EC cells.

Statistical processing was performed using non-parametric Mann-Whitney *U* test in Statistica 6.0 sofftware. Differences were considered statistically significant at *P* < 0.05.

## Results

The obtained results indicate the presence of a heterogeneous population of EC cells bearing on their surface the CD44, CD24, Sca-1 markers and those which could be attributed to accessory-regulatory elements of microenvironment (CD117). The concentrations of cells with these characteristics in total EC pool (group 1.1) are shown in Table 1 and are completely consistent with the previous findings on EC subpopulation composition [28]. Identification of Sca-1 structure virtually in all the EC cells enables to consider it as a versatile marker of this tumor type.

**Table 1:** Total number of cells in peritoneal cavity and their phenotypic parameters to day 7 after administration of total population, CD44^+^ and CD44^–^ EC fractions

Index		Group 1.1. Administration of total population of EC cells (3 × 10^6^ cells/animal)	Group 2.1. Administration of CD44^+^ EC cell fraction (3 × 10^6^ cells/animal)	Group 3.1. Administration of CD44^–^ EC cell fraction (3 × 10^6^ cells/animal)
Concentration of cells with corresponding phenotype, %	CD44^high^	0.17 ± 0.03	0.32 ± 0.05^*^	0.02 ± 0.002^*,**^
	CD44^+^CD24^–^	3.78 ± 0.51	5.65 ± 0.41^*^	3.81 ± 0.28^**^
	CD44^+^CD24^+^	2.89 ± 0.22	4.21 ± 0.50^*^	2.98 ± 0.18^**^
	CD44^–^CD24^+^	5.33 ± 0.64	2.00 ± 0.15^*^	8.50 ± 0.59^*,**^
	Sca-1^+^	90.32 ± 5.33	89.05 ± 4.35	81.30 ± 6.41
	CD117^+^	7.81 ± 0.83	–	3.56 ± 0.06^*^
CD44^high^/CD117^+^ ratio (rel. units)		0.02	–	0.005
TN (×10^7^)		35.80 ± 2.27	826.50 ± 6.53^*^	7.82 ± 0.94^*,**^
MF (rel.units)		116.67 ± 9.60	2755 ± 9.34*	26.07 ± 1.84^*,**^
TD (h)		24.47 ± 2.75	14.70 ± 1.35*	35.71 ± 2.53^*,**^

Notes: the indices are determined to day 7 after administration of total population and isolated CD44^+^ and CD44^–^ EC fractions; *EC* Ehrlich carcinoma, *TN* a total number of EC cells in peritoneal cavity (×10^7^), *MF* multiplicity factor, *TD* time doubling of total EC population in peritoneal cavity

*Differences are statistically significant if compared with group 1.1; **differences are statistically significant if compared with group 2.1 (*P* < 00.5)

The most informative in terms of phenotypic identification of CSCs is CD44 molecule expression, which either itself or in combination with other surface markers are used to isolate this cell population from various tumors, including EC. According to classical notions the differentiation of tumor cells during the breast cancer development  is accompanied by the reduced expression of CD-44 receptor with its gradual disappearance and appearance of the cells expressing the CD24 marker [3].

The candidates for the role of CSCs during EC could be the cells with CD44^high^ phenotype being the part of CD44^+^CD24^–^ - population. This supposition about the dependence of EC on functional activity of a subpopulation of CD44^+^ cells was tested when evaluating the intensity of tumor growth induced by CD44^+^ and CD44^–^ factions and EC total population. Table 1 demonstrates that the highest tumor-inducing activity was inherent to the cells of CD44^+^ fraction. Actually, after administering 3x10^6^ CD44^+^ cells (group 2.1), an absolute number of cells in PC was 23 times higher than after that of total population of EC cells (group 1.1), and 105 times more than when CD44^–^ fraction was administrered (Group 3.1). 

Herewith, there were found changes of not only quantitative but also qualitative compositions of a developing tumor. The fraction of CD44^+^ formed the ascites with a predominant content of CD44^+^ cells, i.e., CD44^high^, CD44^+^CD24^–^, and CD44^+^CD24^+^ cells. Besides, the concentration of CD44^high^ cells was 2 times higher if compared with group 1.1 and 16 times higher in group 3.1. The fraction of CD44^–^, in contrast, formed a tumor which contained more mature cells, namely, those with CD44^–^CD24^+^ phenotype. The very redistribution of subpopulation composition of cells in group 3.1 apparently determined the minimum absolute content of cells in the PC.

Important is the established fact of the presence among the EC cells of a subpopulation with a CD117^+^ marker. The molecule of CD117 is a transmembrane tyrosine kinase receptor. Under normal conditions, it is activated by the corresponding ligand, i.e.- stem cell growth factor (SCGF) [29]. In   oncological pathology the ligandone-dependent activation of the c-KIT receptor occurs, which most often (up to 92% of cases) is a consequence of the c-kit oncogene mutation or is caused by a disordered  mechanisms of regulation of this receptor function [30]. 

Considering CD117^+^ cells as the cells of tumor microenvironment the established fact of dependence of tumor growth intensity on the presence or absence of CD117^+^ cells and their concentration relationships with CD44^high^ cells is logic. As Table 1 shows, when initiating the EC by introducing the total cell population (group 1.1.) in the PC, there were formed 34.80 ± 1.27 × 10^7^ cells at the CD44^high^/CD117^+^ ratio, which was equal to 0.02 relative units.

Tumorigenic potential of CD44^–^ fraction was 4 times lower (group 3.1) that was manifested by a reduced CD44^high^/CD117^+^ ratio to the same extent (4 times) if compared with group 1.1. This change in CD44^high^/CD117^+^ index was mainly due to a decrease in CD44^high^ concentration (in 8.5 times) on the background of the reduced content of CD117^+^ cells (in 2 times) as well.

When evaluating the intensity of ascites growth, generated by CD44^+^ fraction, a significant increase in total number of cells in PC (almost 24-fold if compared to group 1.1) was noted. As well, an important is the excess in two times of CD44^high^ concentration and lack of CD117^+^ cells. In initial material of CD44^+^ fraction (prior to culturing) according to flow cytometric analysis of the data, the content of CD44^high^ cells was 15 times higher than in total population of EC cells (data not presented).

To summarize the mentioned above, we may argue that a crucial role in the initiation of EC is played by CSCs with a high expression rate of CD44 marker (CD44^high^), while one of the most important functions of CD117^+^ subpopulation is a regulation (“restraining”) of tumorigenic activity of CD44^high^ cells. The absence of CD117^+^ cells (group 2.1) seems to multiply the proliferative and differentiation potential of the entire pool of CD44^+^ cells, causing a significant rise in total number of cells in the PC.

The analysis of proliferative potential of a total pool of EC cells and CD44^+^ faction favors this interpretation. It is shown that the multiplication factor (MF) in the overall population cultured in the PC in group 2.1. during 7 days increased almost in 24 times if compared with group 1.1. This was accompanied by a decrease in cell time doubling from 24.47 ± 2.75 h in group 1.1–14.70 ± 1.35 in group 2.1 that may characterize a population of ascites cells grown from CD44^+^ fraction, as more actively proliferating one (Table 1).

To prove the special role of CD44^+^ cells in initiation and maintenance of tumor when administered EC even in minimal doses, it was of interest to comparatively assess the tumorigenic capacity of isolated CD44^+^ and CD44^–^ fractions when administered at various concentrations. It has been found that after the introduction of 3 × 10^6^ cells of total EC population, tumor growth was observed in 100% of animals (10/10) (Table 2). Reducing 10 times the dose of cells administered (3 × 10^5^) resulted in a proportional decrease in absolute number of cells in the PC, tumor developed only in 50% of animals (Table 2). Reducing the administered dose of total EC population of cells down to 3 × 10^4^ did not lead to tumor formation in the PC.

**Table 2:** Tumorigenic activity of total population, isolated CD44^+^ and CD44^–^ EC fractions

Groups	Introducible dose of EC cells	Administration of total population of EC cells	Administration of CD44^+^ EC cell fraction	Administration of CD44^–^ EC cell fraction
Total number of EC cells in the peritoneal cavity (×10^7^) when administering under various concentrations	3 × 10^6^	35.80 ± 1.27	826.51 ± 6.53^*^	7.82 ± 0.94^*^
	3 × 10^5^	3.00 ± 0.25	65.00 ± 1.27^*^	–
	3 × 10^4^	–	13.50 ± 0.31	–
	3 × 10^3^	–	–	–
Number of animals with EC development to day 7 after administration of various concentrations of cells	3 × 10^6^	10/10	10/10	5/10
	3 × 10^5^	5/10	9/10	0/10
	3 × 10^4^	0/10	3/10	0/10
	3 × 10^3^	0/10	0/10	0/10

Note: number of animals with EC development was calculated by considering the number of animals with evident developed EC to day 7 (absolute number of cells in the peritoneal cavity was not less than 35.00 × 10^7^), correlated to the number of animals in each group (*n* = 10)

*Differences are statistically significant as compared with administration of total population of EC cells; (*P* < 0.05)

Initiations of EC by introducing of CD44^+^ cells at concentrations of 3 × 10^6^ and 3 × 10^5^ cells per animal resulted in almost 100% tumor development for both cases. Herewith, tumorigenic potential of CD44^+^ fraction exceeded that of total population of EC cells administered in the same doses (in 23 and 21 times, respectively). Moreover, introduction of 3 × 10^4^ cells of CD44^+^ fraction caused a tumor formation in 33% of animals, while total population of EC cells used in the same dose, did not cause the formation of ascites. With the introduction of 3 × 10^3^ cells of CD44^+^ fraction, no animals with the developed EC have been identified.

Fraction of CD44^–^ cells just in a dose of 3 × 10^6^ was capable of forming tumors in 50% of animals, the number of cells in the PC in this case was 4.5 times less than when introducing the total population and in 105.9 times less than when inducing by CD44^+^ fraction. Thus, the results of this part of research suggest that CSCs are mainly present in the pool of cells with CD44^+^ phenotype. This emphasizes the importance of this subpopulation of cells in initiation and development of EC.

As noted above, identification and inactivation of CSCs is a major theoretical and practical issue of oncology. On this basis, the next task of our study was to investigate the impact of hybrid nanocomplexes designed at the Institute for Scintillation Materials of National Academy of Sciences of Ukraine on the tumorigenic activity of EC cells.

As Table 3 demonstrates an incubation of EC cells with only NPs as a component of hybrid nanocomplexes (option 1) decreased the concentration of CD44^high^ virtually twice if compared to the control and 5 times the content CD44^+^CD24^–^ cells in ascites formed in vivo. The number in it of more differentiated CD44^+^CD24^+^, CD44^–^CD24^+^ cells remained practically unchanged if compared to the control. In this group, there was established reduction of CD117^+^ cells (35%) at a slightly changed content of Sca-1^+^ subpopulation. Based on the data, the inhibition rate of EC growth (59.41 ± 3.45%) in variant 1 was accompanied by a twofold decrease in the concentrations of CD44^high^ cells in comparison with the control that was also reflected in the reduction of CD44^high^/CD117^+^ ratio (Table. 3).

**Table 3:** Change in composition and functional activity of in vivo cultured EC cells after pretreatment with hybrid nanocomplexes

Index		Pre-treatment variants		
		Option 1	Option 2	Control
Concentration of cells with corresponding phenotype, %	CD44^high^	0.09 ± 0.01^*^	0.02 ± 0.001^*, **^	0.17 ± 0.03
	CD44^+^CD24^–^	0.75 ± 0.05^*^	0.40 ± 0.02^*, **,^	3.78 ± 0.51
	CD44^+^CD24^+^	2.75 ± 0.28	9.77 ± 0.62^*, **^	2.89 ± 0.22
	CD44^–^CD24^+^	6.18 ± 0.32	7.85 ± 0.44^*^	5.33 ± 0.64
	Sca-1^+^	81.82 ± 5.28	83.77 ± 5.73	90.32 ± 5.33
	CD117^+^	5.04 ± 0.32	10.47 ± 0.83^*,**^	7.81 ± 0.83
CD44^high^/CD117^+^ ratio (rel. units)		0.018	0.002	0.02
TN (×10^7^)		14.53 ± 0.72^*^	9.06 ± 0.25^*,**^	35.80 ± 2.27
Inhibition rate of EC growth (Ri) (%)		59.41 ± 3.45	74.70 ± 4.38^**^	–

Note: number of animals with EC development was calculated by considering the number of animals with evident EC degree to day 7 (absolute number of cells in the peritoneal cavity was not less than 35.00 × 10^7^), correlated to the number of animals in each group (*n* = 10); *TN* a total number of EC cells in peritoneal cavity (×10^7^)

*Differences are statistically significant as compared with administration of the control; **option 1 (*P* < 0.05)

Pretreatment of EC cells with hybrid nanocomplexes (option 2) reduced almost 10 times the concentration of CD44^high^ and CD44^+^CD24^–^ cells in the developed ascites if compared to the control (Table 3). It should be noted that the concentration of more differentiated CD44^+^CD24^+^ and CD44^–^CD24^+^ cells after this treatment increased slightly if compared to the control. The redistribution pattern of EC subpopulation composition in this option was accompanied with a pronounced enhancement of tumor growth inhibition compared to option 1 (74.70 ± 4.38 and 59.41 ± 3.45%, respectively, *P* < 0.05) that underlined the importance of cholesterol as a targeted compound of antitumor therapy. Pretreatment with hybrid nanocomplexes (option 2) led to maximal reduction there was found a maximum reduction of CD44^high^ /CD117^+^ ratio (10 times) as compared with option 1, that again confirmed a specific role of ratio of these cell subpopulations in the EC growth.

**Fig. 3:**
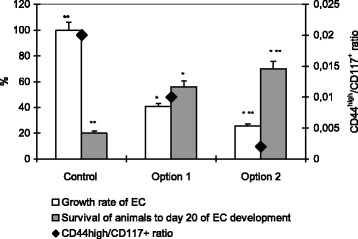
Tumor growth rate of EC, survival of animals and CD44^high^/CD117^+^ ratio after incubation with nanocomplexes. Note: differences are statistically significant as compared with administration of the control (*), option 1 (**) (*P* < 0.05)

For all the types of EC pretreatment, the reduction of CD44^high^/CD117^+^ ratio was accompanied by a decrease in tumor growth rate and increased survival of animals to day 20 of EC development (Fig. 3).

## Discussion

One of the tasks of current oncology is elucidation of the mechanisms of initiation and development of malignant neoplasms. Mandatory participants in these events are the CSCs and so-called accessory-regulatory cells of tumor microenvironment. The variety of functional and structural characteristics of the CSCs in the development of different types of tumors determines the need for their further study. This is facilitated by the expansion of experimental model systems. One of them is the transplantable line of tumor cells of EC.

The elucidation of the peculiarities of this experimental model development, the subpopulation composition of tumor and tumorigenic potential of individual cell populations within the general pool of the EC cells will facilitate the development of new approaches to cancer therapy.

Using the method of phenotypic evaluation of progenitor cells of various levels of differentiation in the tumor focus makes it possible the identifying the stages, dynamics of development and invasiveness of the process. The established fact of heterogeneity of the EC subpopulation composition is important and there has been emphasized the value of CD44^+^ subpopulation in maintaining the growth of this type of tumor.

The most important role in implementing a tumorigenesis is played by an expression rate of the molecule. Indeed, in contrast to leukocytes for adhesion of those normally a low expression rate of CD44 receptor is required, triggering and self-maintenance in CSCs are implemented its much greater density on a cell surface [31].

It is known that CD44-glycoprotein is a hyaluronic acid (HA) receptor, a main component of extracellular matrix. The emerging set of HA-CD44 activates many receptor tyrosine kinases, resulting in activation of PI3K/Akt/ mTOR way [32, 33], which plays the role of a single universal signal transmission mechanism to the translation apparatus and is responsible for the integration of proliferative stimuli.

Among two known CD44-isoforms in normal hematopoietic cells its standard isoform (CD44s) is predominantly expressed [34]. In most malignant tissues there were detected both CD44s and variable isoforms of CD44- molecule (CD44v), resulting from alternative splicing of exons 6-15. Namely alternative splicing leads to a lengthening of CD44-extracellular domain, promoting its greater interaction with HA and tumor metastasis [35]. Due to that the role of CD44^high^ cells in triggering and maintaining the tumorogenesis is clear. It was previously found that a minor subpopulation of CD44^high^ cells had a high proliferative potential and played a critical role in EC developing [20].

In this paper, a special role of CD44^+^ -cells of the EC in initiation and maintenance of the tumor process in the EC under administration even in minimal doses has been shown. CD44^+^ cells were able to form a tumor even at a cell concentration of 100 times lower (10^4^ cells/ mouse) if compared with the introduction of a total EC population (10^6^ cells / mouse). The belonging of tumor cells to the CD44^+^ fraction was also confirmed by the fact that the EC initiation by the fraction of CD44-cells even at a dose of 10^6^ cells / mouse caused the formation of a tumor only in 50% of cases, with an absolute number of cells in the PC 5 times less than in under introduction of a similar amount of the total population of EC and more than 100 times less than after the introduced CD44^+^-fraction.

This is in accordance with the data of Shipitsin M et al. has shown that CD44^+^ and CD24^+^ cells in breast cancer development there are cell populations with different genetic profiles [36]. The research performed by Shipitsin M CD24^+^ cells have been noted to be more differentiated, while more progenitor-like functions are inherent to CD44^+^ cells. The research performed by Shipitsin M CD24^+^ cells have been noted to be more differentiated, while more progenitor-like functions are inherent to CD44^+^ cells. The authors suggest that CD24^+^ cells can be derived from CD44^+^ cells [36]. Fillmore C. and Kuperwasser C. supposed that CD24^+^ population was mainly characterized by less differentiated basal type of breast cancer, and CD44^+^ cells caused the development of luminal form of breast cancer, being more differentiated type of tumor [37].

Analyzing the patterns of tumor development, the classic hypothesis of «seed and soil» looks very actual [38], which postulated that an appropriate microenvironment (soil) is required for optimal growth of tumor cells (CSCs).

Most often the carcinoma-associated fibroblasts (CAFs) act as a tumor stroma in breast cancer and pancreatic cancer [39]. It has been shown that the CAFs, derived from invasive forms of human breast carcinomas, activated much stronger the growth of human breast cancer cell line MCF-7-Ras when administered to immunodeficient mice if compared with normal fibroblasts [9]. This function is implemented by the microenvironment cells due to the secretion by them of cytokines, chemokines and growth factors [10, 40].

Although so far the phenotypic identification of the microenvironment cells for various types of tumor has remained a subject of debate, most often used for this purpose the surface markers of primitive hematopoietic and endothelial cells, including c-kit (CD 117), CD133, VE-cadherin, VEGFR-2 and endoglin are used [41]. In this experimental model the most probable candidate to the role of tumor microenvironment cells is CD 117^+^.

It is known that the c-KIT receptor (CD117^+^) is highly expressed in normal epithelium of the breast and progressively decreases with the development of breast carcinoma in situ and is almost completely lost in invasive breast cancer [42, 43]. Some authors proposed this kind of change in the expression rate of this marker as a possible test to assess the effectiveness of antitumor therapy [44].

Previously, after analysis of the significance of the content ratios for different subpopulations of EC cells when maintaining tumor growth, we proposed to use the CD44^high^/CD117^+^ ratio as a prognostic criterion of tumor development [22].

Adequacy of using this index is confirmed in this study using the applied nanocomposites as therapeutic agents when treating the EC. The inhibition rate of EC growth (59.41 ± 3.45%) when treated with spherical NPs (option 1) was accompanied by a 2-fold decrease if compared to the control in the CD44^high^-cell concentration, which was reflected in the reduced CD44^high^ / CD117^+^ index. The maximum decrease in the CD44^high^/CD117^+^ index (10 times if compared to option 1) was established using the hybrid nanocomplexes for a pre-treatment of EC cells. Thus, many cells of a total pool of EC, but primarily those with the phenotype CD44^high^ and CD117^+^, can be the target of the effect of the studied nanocomplexes (both direct and indirect). A significant decrease in their concentrations in the growing pool of EC after pretreatment with hybrid nanocomplexes clearly coincides with a reduced intensity of tumor growth.

Judging by the decrease in the amount of CD44^high^ as the most potent CSCs forming the entire subsequent series of advanced tumor cells, the main component in manifestation of antitumor effect of the synthesized hybrid nanocomplexes is spherical NPs. Introduction of cholesterol having affinity to tumor cell membranes into composition of hybrid nanocomplexes enhanced an inhibitory activity of NPs. Similar data were obtained by Betker J.L. et al. after analysis of the structure and functioning principles of the membranes of tumor cells. The authors concluded that the incorporation of cholesterol into membranes of tumor cells could be a prerequisite for a targeted delivery of liposomes with therapeutic agents directly into a cell.

Thus, the importance of cooperative interactions of cells with different phenotypic signs in maintaining the EC growth has been proven. The cells with the CD44^high^ phenotype being the part of the population of CD44^+^ CD24^–^ can be considered as CSCs in this model system. The use of new forms of nanocomposites that are capable to bind to CSCs and induce tumor destruction as the EC is a promising direction the treatment of oncopathology.

## Conclusions

1. On the base of the findings of phenotypic assessment and functional potential studies, the Ehrlich carcinoma is a heterogeneous population of tumor cells of varying differentiation extent referred to high and less potent tumor-inducing precursors, as well as the cells composing their microenvironment.
2. A high (tenfold) tumorigenic activity of the EC CD44^+^ cells if compared to CD44^–^ cells was proven. In this pair of comparison, the CD44^+^ cells had a higher potential of generating in PC of CD44^high^, CD44^+^CD24^–^, CD44^+^CD24^+^ cell subpopulations, highlighting the presence of CSCs in a pool of CD44^+^ cells.
3. There was found an ability of the synthesized nanocomplexes based on rare earth orthovanadates and cholesterol to inhibit the growth of CD44^+^ cell pool (CD44^high^, CD44^+^CD24^–^, CD44^+^CD24^+^) that was accompanied by a reduced intensity of EC growth (by 75%) and increased survival of the animal with tumors (in 3.5 times) in comparison with the control.
4. It has been shown that the reduction in tumor growth rate after pretreatment with hybrid nanocomplexes was accompanied with a change in the composition of EC subpopulation that was reflected in a decrease in the CD44^high^/CD117^+^ ratio. This ratio can be offered as one of diagnostic and prognostic tests of the severity and extent of oncology inactivation.

## Abbreviations

BC: Breast cancer
CAFs: Carcinoma-associated fibroblasts
CSCs: Cancer stem cells
DiI: 1,1′-Dioctadecyl-3,3,3′,3′-tetramethylindocarbocyanine perchlorate
EC: Ehrlich carcinoma
HA: Hyaluronic acid
MF: Multiplicity factor
NOD/SCID mice: Nonobese diabetic-severe combined immunodeficiency mice
NPs: Nanoparticles
PC: Peritoneal cavity
Rg: Growth rate of EC
Ri: Inhibition rate of EC growth
SCID mice: Severe combined immunodeficiency mice
TD: Time doubling
